# Efficient AoA-Based Wireless Indoor Localization for Hospital Outpatients Using Mobile Devices

**DOI:** 10.3390/s18113698

**Published:** 2018-10-30

**Authors:** Yanbin Hou, Xiaodong Yang, Qammer H. Abbasi

**Affiliations:** 1 School of Electronic Engineering, Xidian University, Xi’an 710071, China; ybhou@mail.xidian.edu.cn; 2 School of Engineering, University of Glasgow, Glasgow G12 8QQ, UK; Qammer.Abbasi@glasgow.ac.uk

**Keywords:** hospital wayfinding, indoor localization, wireless localization, Wi-Fi localization, angle of arrival (AoA), location-based services (LBS)

## Abstract

The motivation of this work is to help outpatients find their corresponding departments or clinics, thus, it needs to provide indoor positioning services with a room-level accuracy. Unlike wireless outdoor localization that is dominated by the global positioning system (GPS), wireless indoor localization is still an open issue. Many different schemes are being developed to meet the increasing demand for indoor localization services. In this paper, we investigated the AoA-based wireless indoor localization for outpatients’ wayfinding in a hospital, where Wi-Fi access points (APs) are deployed, in line, on the ceiling. The target position can be determined by a mobile device, like a smartphone, through an efficient geometric calculation with two known APs coordinates and the angles of the incident radios. All possible positions in which the target may appear have been comprehensively investigated, and the corresponding solutions were proven to be the same. Experimental results show that localization error was less than 2.5 m, about 80% of the time, which can satisfy the outpatients’ requirements for wayfinding.

## 1. Introduction

With the popularity of mobile devices, such as smartphones, smartwatches, tablets, as well as some wearable devices, wireless localization has spawned numerous location-based services (LBS) to facilitate our daily life in the fields of emergency services, navigation, healthcare, geofence, social networking, entertainment/gaming, asset tracking, etc. [1,2,3,4,5,6]. For example, smartphone users can easily plan their trips using Google Maps and order a nearby taxi. The accurate location is mainly provided by the global positioning system (GPS). However, GPS signal is degraded too severely to be received in indoor environments. Many methods have been developed to meet the increasing demand for indoor LBS applications [7,8,9]. These wireless indoor localization methods and systems can be roughly divided into two main categories: range-based schemes and range-free schemes.

Range-based schemes first measure the distances between the target and at least three reference points, and then determine the target’s position by triangulation or trilateration, using the least square method. The distances are indirectly measured by interpreting some physical parameters obtained from wireless signals, such as time of flight (ToF), time of arrival (ToA), time difference of arrival (TDoA), and received signal strength indication (RSSI) [10,11,12,13,14,15,16]. ToF, ToA, and TDoA are very sensitive to timing error, thus, relying on highly synchronized timers. Furthermore, they usually need work under line of sight (LOS) conditions to get accurate ranging. RSSI is used to figure out the distance between the transmitter and the receiver with log-normal distance path loss (LDPL) model [17]. However, indoor RSSI is subject to temporal and spatial fluctuations because of the multipath effect, usually providing a coarse-grained positioning. The RSSI is not robust to noise because signal attenuates with propagation distance while noise usually does not.

Range-free schemes can be further subdivided into a distance vector hop (DV-hop), proximity detection, fingerprint matching, and angle of arrival (AoA)-based methods [18,19,20,21,22,23,24]. In a wireless sensor network (WSN), hop counts are utilized to estimate the positions of sensors with initially-unknown location information, by using a priori knowledge of the absolute positions of a few sensors (named as anchor nodes) and inter-sensor measurements, such as distance and bearing [25,26,27]. In the proximity detection method, Bluetooth low energy (BLE) beacons are deployed to periodically broadcast advertising messages, which contain their positions and other information [19,28,29]. The nearby mobile devices detect the signal from the beacon and calculate the approximate distance to the beacon. Thus, it needs to deploy a mass of beacons to obtain an accurate location. Wi-Fi can be used in a similar way as the BLE beacons, but requires external power sources and more setup costs. Fingerprint matching is a pattern recognition approach. During the training phase, signal features of all regions of interest are collected to build a fingerprint database [22]. Then, the localization is a process of matching the measured fingerprint, at an unknown location, with those in the database. Finally, the location corresponding to the best-fitted fingerprint is returned. Obviously, fingerprint matching is time-consuming and it is labor-intensive to build and maintain the database. AoA, also known as direction of arrival (DoA), refers to the angle of incidence at which radio signals from a transmitter arrive at the receiver. Given two AoAs and their receivers’ positions, the two-dimensional coordinates of the transmitter can be determined by triangulation. Accurate AoA can only be obtained by directional antennas or an antenna array, which makes the receiver complex and expensive.

Since the RSSI can be easily obtained by common mobile terminals, such as smartphones, Wi-Fi devices, Bluetooth beacons, ZigBee sensors, and so on, it is widely used to study wireless localization in range-based and range-free schemes. The fundamental drawback of RSSI is that it only works well in LOS situations. Unlike the RSSI, which is an aggregated value of all of the subcarriers’ amplitudes, the channel state information (CSI) estimates the channel, on each subcarrier, in the frequency domain. The CSI is to RSSI what a rainbow is to a sunbeam, so it can depict a multipath propagation to some extent and possesses more stable and fine-grained localization capability. The CSI can estimate the AoA and the ToF for multipath components under non line of sight (NLOS) conditions [30]. However, it needs to modify the firmware or update the hardware on available commercial devices to obtain the CSI data, which is difficult to implement on handheld devices, like smartphones. More details about the RSSI and the CSI can be found in Reference [31]. It is worth noting that hybrid methods are increasingly introduced in order to enhance the localization performance, such as the RSSI/DV-hop localization algorithm for the WSN, using fingerprinting method to improve the AoA-based ranging technique, the RSSI/AoA-based target localization and tracking, and so on [27,32,33,34].

In this paper, we propose an efficient AoA-based wireless indoor localization method for the outpatients’ wayfinding, in hospital, using mobile devices, such as tablets, smartphones, smartwatches, and so on. The Wi-Fi access points (APs) with antenna arrays are hung on the ceiling, so that radio signals can reach targets, along straight lines, in most situations. When users inquire about their locations using mobile devices like smartphones, nearby APs will timely return the positions of the APs and the AoAs pointing to the users’ current positions. Then the smartphones efficiently figure out the users’ locations, through geometric calculations. Both the Wi-Fi APs and the smartphones are off-the-shelf. Experimental results show that indoor localization error is less than 2.5 m, about 80%, of the time in LOS environments.

The contributions of this work are: (1) The proposed AoA-based algorithm is to employ only two Aps, at a time, for target localization. (2) The solutions are proven to be in the same form, after all the possible positions where the target may appear are investigated, comprehensively. (3) The calculation only involves a simple tangent function and basic arithmetic operations, which can be efficiently performed on a handheld mobile device.

The rest of this paper is organized as follows. First, Section 2 reviews the related work. Then, Section 3 introduces the design method and localization algorithm, in detail. Section 4 gives the experimental results. Finally, Section 5 concludes this work and discusses future work.

## 2. Related Work

AoA can be estimated by finding the maximum or minimum signal strength, during the rotation of a directional or a non-ideal omnidirectional monopole antenna [35,36,37,38], or when measuring the phase difference of received signals in an antenna array [39,40,41,42]. 

For the directional antenna, it needs an additional mechanical unit to rotate, and takes a long period of time to sample the 360° data [43]. So, it is impractical to be equipped in a handheld platform, such as smartphones, smartwatches, tablets, and so on. Zhang et al. proposed an alternative approach to emulate the functionality of a directional antenna, and developed an outdoor AP localization system, called Borealis [44]. In Borealis, a user holds a smartphone in the hand and rotates the body by 360°, a group of RSSI data is recorded and further processed to estimate the AP direction. Borealis produces 30° angular error in simple LOS environment, up to 50° in complex LOS, and 65° in NLOS environments. SpinLoc adopts a similar localization principle as Borealis, except that it replaces the RSSI by the CSI for indoor scenarios [45]. SpinLoc can produce a median error of 20°, offering localization accuracies in order of 6.5 m, with 4 Aps, and up to 5 m, with more APs. Jiang et al. proposed an AoA Localization with RSSI Difference (ALRD) system to estimate the AoA by comparing the RSSI values, about the target, received from two perpendicularly-oriented directional antennas, at the beacon nodes [37]. ALRD has an average localization error of 1.24 m at the CDF (Cumulative Distribution Function) = 50% in a 10 × 10 m indoor area. Although the ALRD need not rotate the directional antennas to collect data, it has to rebuild a new fingerprint database of the RSSI values when the target changes.

Antenna array is another common approach to estimate the AoA, by measuring the phase difference between the received signals of the two antennas. The phase difference is caused by the time difference of the signal that arrives in the different antennas, and is essentially determined by the incident angle of the incoming signal. Amundson et al. employed radio interferometric technique to determine the bearing from an anchor node, with an antenna array, to a distant target node, achieving an accuracy of about 3° [39]. Based on the above radio interferometric AoA estimation, Amundson et al. designed a localization and navigation system, called the TripNav, which could provide an average position and heading accuracy of 0.95 m and 4.75°, when traveling indoor at a speed of 1 m/s [23]. However, it requires an accurate time synchronization between the anchor node and the target node, on the order of microseconds. Chen et al. designed a low-cost and flexible antenna array for the AoA estimation, using modular, commercial-off-the-shelf, software-defined radios (SDRs) [42]. The prototype system still needs an additional clock module to synchronize the connected SDRs, for measuring the phase difference of arrival (PDoA), and offers a localization error less than 3 m, in the open field test. In the indoor location system, called the ArrayTrack, a custom FPGA (Field Programmable Gate Array) hardware with sixteen antennas was designed, and a multi-path suppression algorithm, named MUSIC (Multiple Signal Classification), was applied for the AoA estimation [40]. ArrayTrack can pinpoint forty-one clients, over an indoor office environment, to within 0.23 m median accuracy. Tzur et al. used a commercial Intel 5300 Wi-Fi NIC (Network Interface Card) with two receiving antennas to develop a practical method for identifying the AoA of a Wi-Fi AP [46]. In a realistic indoor environment, they are able to acquire the AoA, with a median error of 8–15°. SpotFi uses a single NIC with three antennas, combining the CSI values across the subcarriers and the antennas to jointly estimate both the AoA and the ToA of each path [47]. The joint estimation procedure enables the SpotFi to provide a similar AoA accuracy that is comparable to systems that require twice as many antennas. Under the LoS conditions, SpotFi can achieve a median error of 5°. Gjengset et al. used multiple NICs and automatic calibration to achieve a median location accuracy of 0.9 m [41].

## 3. Methodology

In this section, we first introduce the basic idea of our method. And then we give the detailed description of all possible cases in which the target will appear with regards to the Wi-Fi APs. Finally, we show how to deal with the case in which a target can be accessed by several pairs of APs.

### 3.1. AoA-Based Localization with Two Known APs

**Figure 1:**
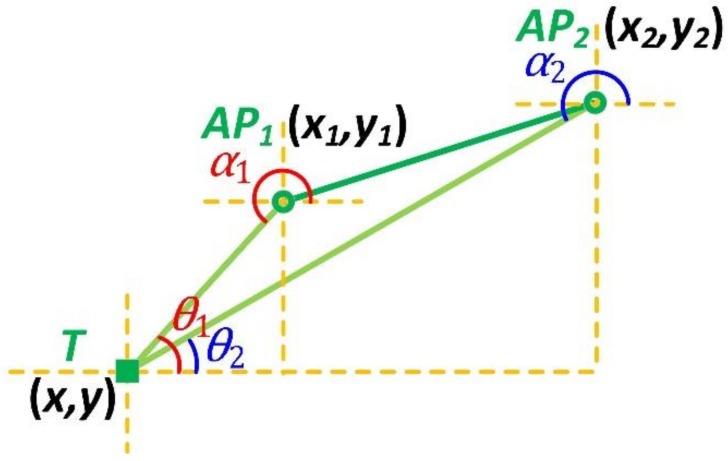
The angle of arrival (AoA)-based localization with two known access points (APs).

We plot Figure 1 to interpret the AoA-based localization with two known APs. For the convenience of description, all positions and angles in this paper are expressed in the two-dimensional Cartesian coordinate system. And the AoA value is defined to be positive if it is counter-clockwise and negative otherwise. In order to avoid confusion, we further define that the AoA specifically refers to the angle of incidence at which the radio signal travels from the transmitter to the receiver, along a straight line.

As shown in Figure 1, two Wi-Fi APs are located at $\left(x_{1},y_{1}\right)$ and $\left(x_{2},y_{2}\right)$, labelled as $AP_{1}$ and $AP_{2}$, respectively. Without the loss of generality, let $x_{1}\neq x_{2}$ and $y_{1}\neq y_{2},$ here. An arbitrary mobile target, labelled as T, is needed to be localized, by determining its coordinates (x,y) with the two known APs and the corresponding AoAs. From the standpoint of the target, the AoAs related to $AP_{1}$ and $AP_{2}$ are $\theta_{1}$ and $\theta_{2}$, respectively, but if the AoAs are measured by $AP_{1}$ and $AP_{2}$, they will be $\alpha_{1}$ and $\alpha_{2}$.

As stated above, the undetermined coordinates (x,y) of target T can be formulated by the following tangent functions

(1) $$\;\{\begin{array}{c} y_{1}-y=\left(x_{1}-x\right)\tan\theta_{1}\\ y_{2}-y=\left(x_{2}-x\right)\tan\theta_{2} \end{array}\;$$

The solution to Equation (1) is

(2) $$\;\{\begin{array}{c} x=\frac{y_{2}-y_{1}+x_{1}\tan\theta_{1}-x_{2}\tan\theta_{2}}{\tan\theta_{1}-\tan\theta_{2}}\;\;\;\;\;\;\;\;\\ y=y_{2}-\frac{\left(x_{2}-x_{1}\right)\tan\theta_{1}-\left(y_{2}-y_{1}\right)}{\tan\theta_{1}-\tan\theta_{2}}\tan\theta_{2} \end{array}\;$$

It is easily observed in Figure 1 that two pairs of the AoAs, named as $\alpha_{1}$ and $\alpha_{2}$, $\theta_{1},$ and $\theta_{2}$, have the following relations:

(3) $$\;\{\begin{array}{c} \theta_{1}=\alpha_{1}-180{}^{\circ}\\ \theta_{2}=\alpha_{2}-180{}^{\circ} \end{array}\;$$

Considering the periodicity of the tangent function, we can rewrite the solution to Equation (1) by replacing $\theta_{1}$ and $\theta_{2}$ with $\alpha_{1}$ and $\alpha_{2}$

(4) $$\;\{\begin{array}{c} x=\frac{y_{2}-y_{1}+x_{1}\tan\alpha_{1}-x_{2}\tan\alpha_{2}}{\tan\alpha_{1}-\tan\alpha_{2}}\;\;\;\;\;\;\;\;\\ y=y_{2}-\frac{\left(x_{2}-x_{1}\right)\tan\alpha_{1}-\left(y_{2}-y_{1}\right)}{\tan\alpha_{1}-\tan\alpha_{2}}\tan\alpha_{2} \end{array}\;$$

If APs are installed in a line, the solution will have a more concise form. For example, when the APs are in the same row, which means $y_{1}=y_{2}$, solutions in Equation (4) will be simplified as

(5) $$\;\{\begin{array}{c} x=\frac{x_{1}\tan\alpha_{1}-x_{2}\tan\alpha_{2}}{\tan\alpha_{1}-\tan\alpha_{2}}\;\;\;\;\;\;\\ y=y_{2}-\frac{\left(x_{2}-x_{1}\right)\tan\alpha_{1}\tan\alpha_{2}}{\tan\alpha_{1}-\tan\alpha_{2}} \end{array}\;$$

Unlike the least-squares solution, by using a complex matrix equation in Reference [48], the solutions in Equations (2), (4), and (5) show that our method can efficiently figure out the target’s position, through some relatively simple trigonometric calculations.

### 3.2. All Possible Positions of the Target around the APs

In this section, we will investigate how to figure out all the possible positions of a mobile target, around the Aps, in practice. When a user holds a mobile device (such as smartphone, smartwatch, tablet, etc.) to wander in a shopping mall, workshop, or hospital, he or she will appear in any position randomly. Different positions will get different AoAs. We group these positions into normal and special situations, according to the AoAs. In a normal situation, the tangent functions of the AoAs have non-zero values, while in the special situation they have zero or infinite values. 

**Figure 2:**
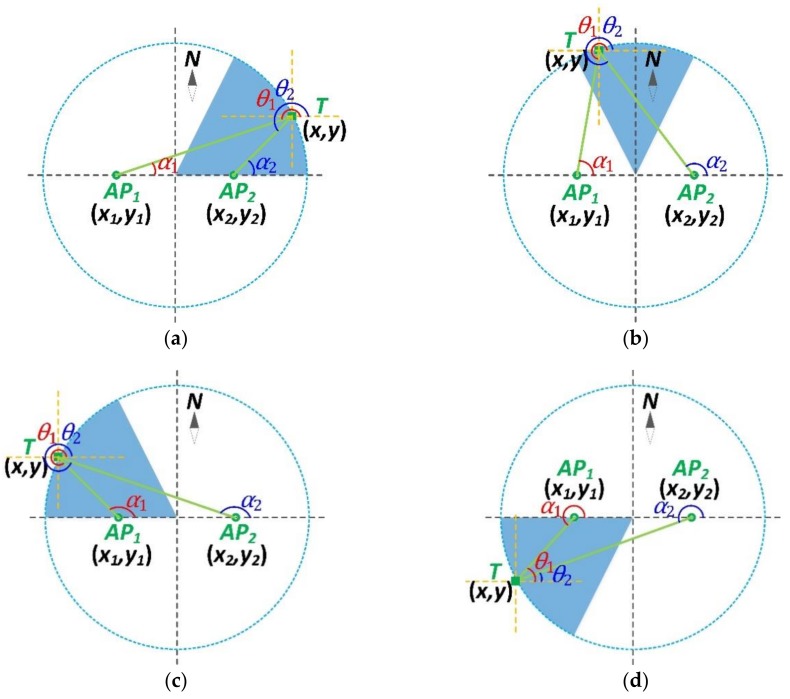

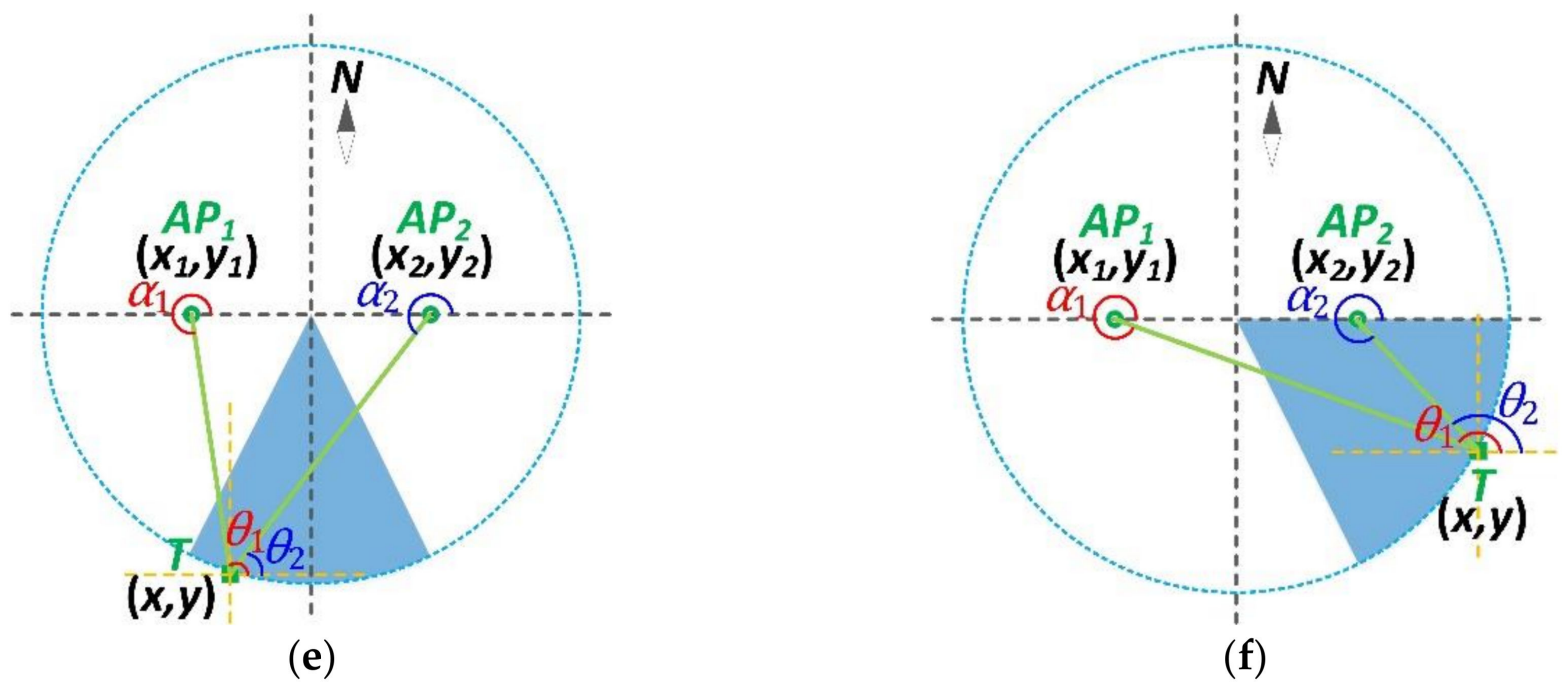
Six normal cases of the target’s position. (**a**) Case 1, (**b**) Case 2, (**c**) Case 3, (**d**) Case 4, (**e**) Case 5, (**f**) Case 6.

As shown in Figure 2, the normal situation is summarized into six cases. In order to be more intuitive, let $y_{1}=y_{2}$, so that $AP_{1}$ and $AP_{2}$ are aligned in the same row.

Case 1: $\alpha_{1},\;\alpha_{2}\in \left(0{}^{\circ},90{}^{\circ}\right)$, as shown in Figure 2a, the equation can be written as

(6) $$\;\{\begin{array}{c} y-y_{1}=\left(x-x_{1}\right)\tan\alpha_{1}\\ y-y_{2}=\left(x-x_{2}\right)\tan\alpha_{2} \end{array}\;$$

The solution to Equation (6) is

(7) $$\;\{\begin{array}{c} x=\frac{x_{1}\tan\alpha_{1}-x_{2}\tan\alpha_{2}}{\tan\alpha_{1}-\tan\alpha_{2}}\;\;\;\;\;\;\\ y=y_{2}-\frac{\left(x_{2}-x_{1}\right)\tan\alpha_{1}\tan\alpha_{2}}{\tan\alpha_{1}-\tan\alpha_{2}} \end{array}\;$$

Considering the geometric relations of $\theta_{1}=\alpha_{1}+180{}^{\circ}$ and $\theta_{2}=\alpha_{2}+180{}^{\circ}$, and the periodicity of the tangent function, we can get another form of the solution

(8) $$\;\{\begin{array}{c} x=\frac{x_{1}\tan\theta_{1}-x_{2}\tan\theta_{2}}{\tan\theta_{1}-\tan\theta_{2}}\;\;\;\;\;\;\\ y=y_{2}-\frac{\left(x_{2}-x_{1}\right)\tan\theta_{1}\tan\theta_{2}}{\tan\theta_{1}-\tan\theta_{2}} \end{array}\;$$

Case 2:$\;\alpha_{1}\in \left(0{}^{\circ},90{}^{\circ}\right),\;\alpha_{2}\in \left(90{}^{\circ},180{}^{\circ}\right)$, as shown in Figure 2b, the equation can be written as

(9) $$\;\{\begin{array}{c} y-y_{1}=\left(x-x_{1}\right)\tan\alpha_{1}\;\;\;\;\;\;\;\;\\ y-y_{2}=\left(x_{2}-x\right)\tan\left(180{}^{\circ}-\alpha_{2}\right) \end{array}\;$$

As $\tan\left(180{}^{\circ}-\alpha_{2}\right)=-\tan\alpha_{2}$, Equation (9) can be rewritten as

(10) $$\;\{\begin{array}{c} y-y_{1}=\left(x-x_{1}\right)\tan\alpha_{1}\\ y-y_{2}=\left(x-x_{2}\right)\tan\alpha_{2} \end{array}\;$$

Equation (10) is the same as Equation (6), so their solutions are also the same. 

Similarly, since $\theta_{1}=\alpha_{1}+180{}^{\circ}$ and $\theta_{2}=\alpha_{2}+180{}^{\circ}$, we can also obtain the same solution as in Equation (8).

Case 3: $\alpha_{1},\;\alpha_{2}\in \left(90{}^{\circ},180{}^{\circ}\right)$, as shown in Figure 2c, the equation can be written as

(11) $$\;\{\begin{array}{c} y-y_{1}=\left(x_{1}-x\right)\tan\left(180{}^{\circ}-\alpha_{1}\right)\\ y-y_{2}=\left(x_{2}-x\right)\tan\left(180{}^{\circ}-\alpha_{2}\right) \end{array}\;$$

As $\tan\left(180{}^{\circ}-\alpha_{1}\right)=-\tan\alpha_{1}$ and $\tan\left(180{}^{\circ}-\alpha_{2}\right)=-\tan\alpha_{2}$, Equation (11) can be rewritten as

(12) $$\;\{\begin{array}{c} y-y_{1}=\left(x-x_{1}\right)\tan\alpha_{1}\\ y-y_{2}=\left(x-x_{2}\right)\tan\alpha_{2} \end{array}\;$$

Equation (12) is the same as Equation (6), so their solutions are also the same. 

Similarly, since $\theta_{1}=\alpha_{1}+180{}^{\circ}$ and $\theta_{2}=\alpha_{2}+180{}^{\circ}$, we can also obtain the same solution as in Equation (8).

Case 4: $\alpha_{1},\;\alpha_{2}\in \left(180{}^{\circ},270{}^{\circ}\right)$, as shown in Figure 2d, the equation can be written as

(13) $$\;\{\begin{array}{c} y_{1}-y=\left(x_{1}-x\right)\tan\theta_{1}\\ y_{2}-y=\left(x_{2}-x\right)\tan\theta_{2} \end{array}\;$$

The solution to Equation (13) is

(14) $$\;\{\begin{array}{c} x=\frac{x_{1}\tan\theta_{1}-x_{2}\tan\theta_{2}}{\tan\theta_{1}-\tan\theta_{2}}\;\;\;\;\;\;\\ y=y_{2}-\frac{\left(x_{2}-x_{1}\right)\tan\theta_{1}\tan\theta_{2}}{\tan\theta_{1}-\tan\theta_{2}} \end{array}\;$$

Considering the geometric relations of $\theta_{1}=\alpha_{1}-180{}^{\circ}$ and $\theta_{2}=\alpha_{2}-180{}^{\circ}$, and the periodicity of the tangent function, we can get another form of the solution

(15) $$\;\{\begin{array}{c} x=\frac{x_{1}\tan\alpha_{1}-x_{2}\tan\alpha_{2}}{\tan\alpha_{1}-\tan\alpha_{2}}\;\;\;\;\;\;\\ y=y_{2}-\frac{\left(x_{2}-x_{1}\right)\tan\alpha_{1}\tan\alpha_{2}}{\tan\alpha_{1}-\tan\alpha_{2}} \end{array}\;$$

Case 5: $\alpha_{1}\in \left(270{}^{\circ},360{}^{\circ}\right),\;\alpha_{2}\in \left(180{}^{\circ},270{}^{\circ}\right)$, as shown in Figure 2e, the equation can be written as

(16) $$\;\{\begin{array}{c} y_{1}-y=\left(x-x_{1}\right)\tan\left(180{}^{\circ}-\theta_{1}\right)\\ y_{2}-y=\left(x_{2}-x\right)\tan\theta_{2}\;\;\;\;\;\;\;\; \end{array}\;$$

As $\tan\left(180{}^{\circ}-\theta_{1}\right)=-\tan\theta_{1}$, Equation (16) can be rewritten as

(17) $$\;\{\begin{array}{c} y_{1}-y=\left(x_{1}-x\right)\tan\theta_{1}\\ y_{2}-y=\left(x_{2}-x\right)\tan\theta_{2} \end{array}\;$$

Equation (17) is the same as Equation (13), so their solutions are also the same. 

Similarly, since $\theta_{1}=\alpha_{1}-180{}^{\circ}$ and $\theta_{2}=\alpha_{2}-180{}^{\circ}$, we can also obtain the same solution as Equation (15). 

Case 6: $\alpha_{1},\;\alpha_{2}\in \left(270{}^{\circ},360{}^{\circ}\right)$, as shown in Figure 2f, the equation can be written as

(18) $$\;\{\begin{array}{c} y_{1}-y=\left(x-x_{1}\right)\tan\left(180{}^{\circ}-\theta_{1}\right)\\ y_{2}-y=\left(x-x_{2}\right)\tan\left(180{}^{\circ}-\theta_{2}\right) \end{array}\;$$

As $\tan\left(180{}^{\circ}-\theta_{1}\right)=-\tan\theta_{1}$ and $\tan\left(180{}^{\circ}-\theta_{2}\right)=-\tan\theta_{2}$, Equation (18) can be rewritten as

(19) $$\;\{\begin{array}{c} y_{1}-y=\left(x_{1}-x\right)\tan\theta_{1}\\ y_{2}-y=\left(x_{2}-x\right)\tan\theta_{2} \end{array}\;$$

Equation (19) is the same as Equation (13), so their solutions are also the same. 

Similarly, since $\theta_{1}=\alpha_{1}-180{}^{\circ}$ and $\theta_{2}=\alpha_{2}-180{}^{\circ}$, we can also obtain the same solution as Equation (15). 

So far, we have investigated all the six cases in a normal situation. No matter where the target locates around the two APs and no matter which pairs of the AoAs are used, the results show that the equations are essentially consistent and that solutions are the same.

**Figure 3:**
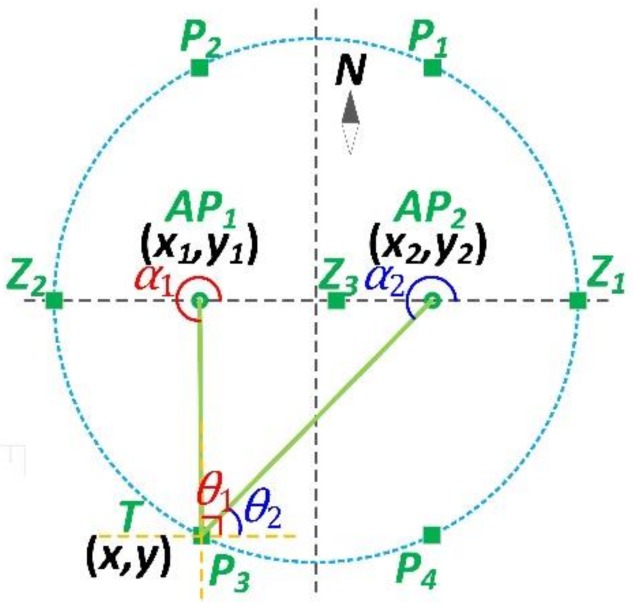
Special cases of the target’s position.

Compared with the normal situation, the special situation consists of blind and pole points, as shown in Figure 3. 

Blind points refer to the positions in which the tangent values of the AoAs are zero. According to the properties of the tangent function, when an angle is 0° or integer multiples of 180°, its corresponding tangent value is zero. Thus the positions in the same row with the APs, such as $Z_{1}$, $Z_{2}$, and $Z_{3}$, are the blind points.

Pole points refer to the positions in which either of the tangent values of the AoAs is infinity. Mathematically, when an angle is integer multiples of 90°, its corresponding tangent value is infinity. Thus, the positions in the same column with either of the APs, such as $P_{1}$, $P_{2}$, $P_{3}$, and $P_{4}$, are pole points.

Obviously, any of blind points fails to constitute a triangle, along with $AP_{1}$ and $AP_{2}$. Thus, it cannot figure out the coordinates of the blind points using the tangent functions. Users should avoid these positions when adopting the AoA-based localization algorithm. As for the pole points, it is easier to calculate the target’s coordinates. Taking $P_{3}$ for example, we can directly obtain $x=x_{1}$ by measuring $\theta_{1}=90{}^{\circ}$, so that we only need to figure out the vertical coordinate y. Using the tangent function, we have

(20) $$\;y_{2}-y=\left(x_{2}-x\right)\tan\theta_{2}\;$$

Thus, $y=y_{2}-\left(x_{2}-x\right)\tan\theta_{2}$. Along with $x=x_{1}$, we get the position of $P_{3}$ as follows

(21) $$\;\{\begin{array}{c} x=x_{1}\;\;\;\;\;\;\;\;\;\;\;\;\;\;\;\;\\ y=y_{2}-\left(x_{2}-x_{1}\right)\tan\theta_{2} \end{array}\;$$

### 3.3. Roaming Strategy

**Figure 4:**
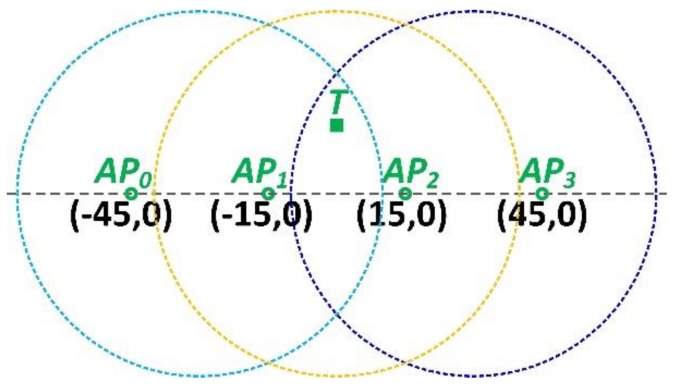
The roaming strategy.

As shown in Figure 4, the target can be accessed by the four APs. There are six combinations in total for choosing two out of four APs, which are $AP_{0}$ and $AP_{1}$, $AP_{1}$ and $AP_{2}$, $AP_{1}$ and $AP_{3}$, $AP_{0}$ and $AP_{2}$, $AP_{0}$ and $AP_{3}$, and $AP_{2}$ and $AP_{3}$, respectively.

Although it is able to localize the target with the aid of any pair of the APs. For the sake of accuracy and efficiency, we adopt a simple but effective roaming strategy to deal with such uncertainty or collision. When there are more than two APs available for estimating the target’s AoAs, the two with the highest RSSI are used. As shown in Figure 4, the four APs are supposed to be of the same model and the same configuration. It is obvious that $AP_{1}$ and $AP_{2}$ have the higher RSSI measurements for the radio from target, compared with the other combinations, because the target is much closer in distance to $AP_{1}$ and $AP_{2}$.

## 4. Experiments and Results

In this section, we first introduce the experimental setup and then carry out the performance evaluation.

### 4.1. Experimental Setup

In our experiment, the AoAs were measured with Cisco Aironet^TM^ 4800 Wi-Fi Aps (Cisco Systems Inc., San Jose, CA, USA), and were retrieved offline from the Cisco Connected Mobile Experiences (CMX) software. Aironet 4800 series APs were designed for the multiple-purpose applications, equipped with up to four radios: Flexible 2.4 GHz or 5 GHz for the Wi-Fi, dedicated 5 GHz for Wi-Fi, 2.4 GHz, and 5 GHz for the Hyperlocation, 2.4 GHz for the BLE. Hyperlocation is an enhanced location solution developed by Cisco, consisting of a few hardware instruments and software components. The workflow in Hyperlocation was: 4800 AP listened to the connected mobile devices through a circular array of sixteen antennas and created packets to record the measured phase differences; then, these packets were sent to the CMX, at a rate of about 1 packet per second per AP, via the WLC (Wireless Local Area Network Controller); finally, the CMX extracted the AoA information from the phase differences and performed the localization. It took 10–20 s to complete one localization, based on the AoA information, and provided a localization accuracy of 1–3 m, in the LOS conditions. Hyperlocation could also provide a real-time location service, relying only on the RSSI, if connected mobile device did not support the OFDM (Orthogonal Frequency Division Multiplexing) technique, but at the cost of a much poor accuracy. More details on the Aironet 4800 series AP and the Hyperlocation Solution can be found on the data sheet and the Cisco website [49,50]. 

**Figure 5:**
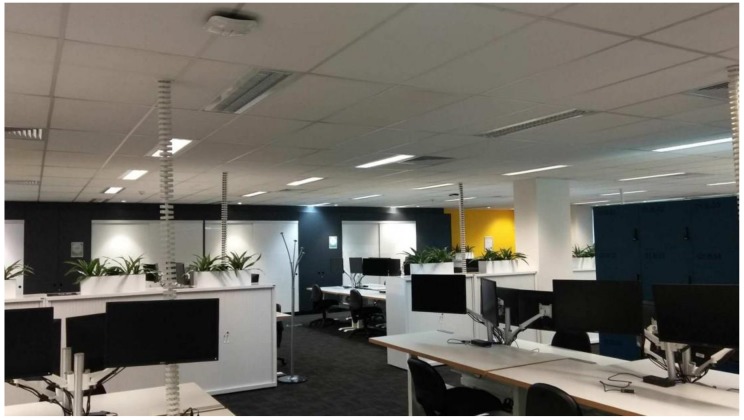
The indoor test environment.

Two 4800 APs were hung from a 3 m high ceiling, in an open laboratory, and an Android smartphone (Coolpad C106 Coolpad Group Limited, Shenzhen, China), with dual-band radio transceivers, supporting the IEEE 802.11a/b/g/n/ac standards) was used as the target. As shown in Figure 5, the laboratory was furnished with a few desks, chairs, and PCs to simulate a hospital waiting hall. The due east of the compass in the smartphone was defined as the positive half of the horizontal axis, and the angle was defined to be positive if it was counter-clockwise and negative, otherwise. In order to ensure the signal strength, the smartphone was connected to the 2.4 GHz Wi-Fi. When a user needed to inquire the location, via a smartphone, the user stopped walking and waited to receive the APs’ coordinates and their corresponding AoA measurements. With these parameters, the smartphone pinpointed the user’s current coordinates, as described in Section 3.

### 4.2. The Impact of the Distance and Direction

**Figure 6:**
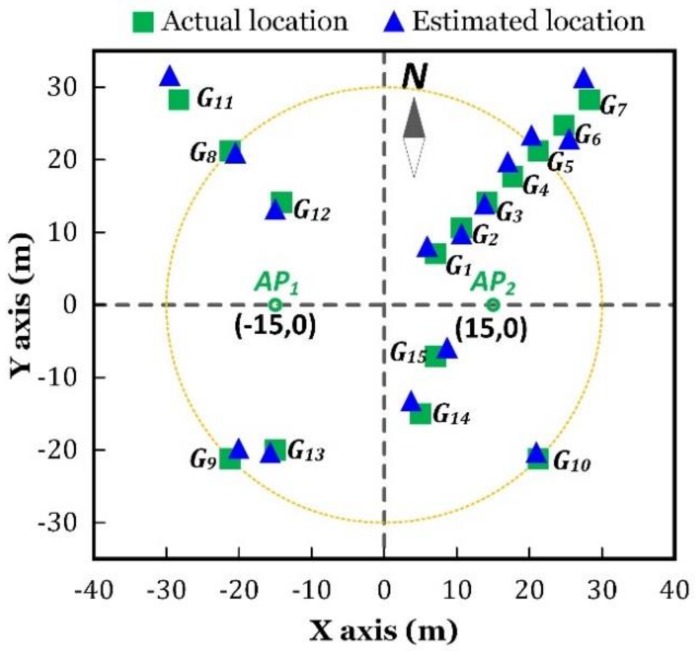
Indoor localization results.

In order to investigate the impact of distance and direction on the localization accuracy, we chose a series of positions as the ground truth, which are marked with green squares in Figure 6. 

As shown in Figure 6, $G_{1}$ to $G_{7}$ were located in the same direction, to the center of $AP_{1}$ and $AP_{2}$, with a distance increasing from 10 m to 40 m. $G_{5}$, $G_{8}$, $G_{9}$, and $G_{10}$ were located in the same distance to the center of $AP_{1}$ and $AP_{2}$, and they were in symmetry, with respect to the horizontal or the vertical axis. $G_{11}$ to $G_{15}$ were random test positions. 

**Figure 7:**
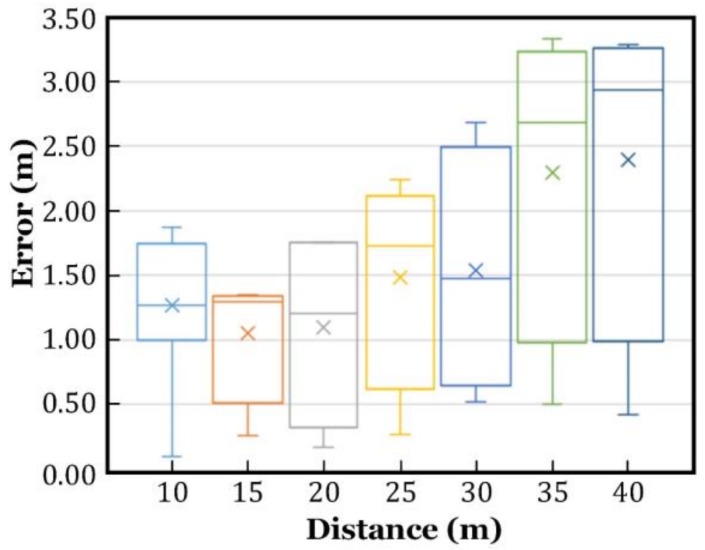
Indoor localization errors.

The localization results are illustrated in Figure 6. The green squares represent the actual locations of the test positions, and the blue triangles are the estimated locations. Localization was performed for eight times, in every test position, to obtain the statistical outcome, as plotted in Figure 7. One group of the results are listed in Table 1, in which the localization error is the Euclidean distance between the estimated and the actual locations, which was obtained by the following.

(22) $$\Delta d=\sqrt{{\left(x_{E}-x_{A}\right)}^{2}+{\left(y_{E}-y_{A}\right)}^{2}}\;$$

where, $\left(x_{E},y_{E}\right)$ represented the coordinates of the estimated location, and $\left(x_{A},y_{A}\right)$ were the coordinates of the actual location.

Figure 7 shows that the localization accuracy decreased as the distance increased, despite the fact that the minimum, maximum, median, and the mean values of the location errors fluctuated. However, it is worth noting that the median and mean location errors did not increase monotonically. For example, most estimations at the distance of 15 m or 20 m, had less location errors than those at the distance of 10 m. This phenomenon was not solely caused by a measurement deviation, but mainly resulted from the ignored fact that the target and the APs were located in a three-dimensional space, rather than on a two-dimensional plane. In other words, the APs provided the AoA measurements in a three-dimensional space, which were mixture of the azimuth and elevation. In our calculations, they were regarded as the azimuth angles. From a mathematical point of view, the farther the distance between the target and APs, the smaller the error will be when the three points of different heights were approximated to be on the XOY plane. 

As shown in Table 1, it was obvious that location accuracy depended on the AoAs. Another noteworthy phenomenon was that the $G_{5}$, $G_{8}$, $G_{9}$, and $G_{10}$ were located, symmetrically, at the same distance to the center of the $AP_{1}$ and $AP_{2}$, but their corresponding AoAs errors and location errors were quite different. This was mainly related to the coverage or directionality of the antenna array. Theoretically, an antenna should be isotropic with a uniform spherical coverage. In fact, no antenna could radiate radio waves, equally, in all directions. In addition, the posture that a user holds the smartphone in, to communicate with the APs and the surroundings, contributed to the deviation in the location errors.

### 4.3. Comparison with RSSI Range-Based Method

**Figure 8:**
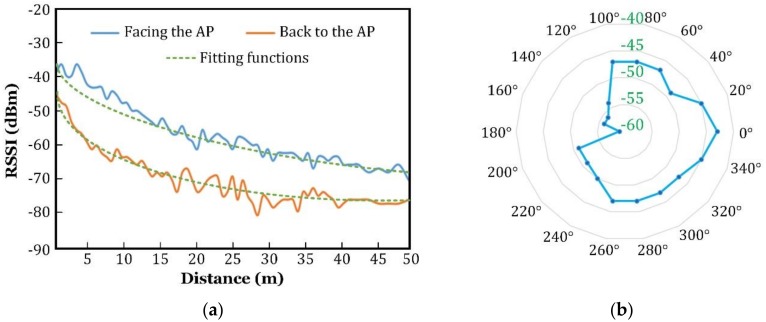
The measured RSSI profiles. (**a**) When the user holds the smartphone and walks; (**b**) when the user holds smartphone and spins.

We repeated the localization using the RSSI range-based method in the positions $G_{1}$ to $G_{7}$, in order to make a comparison with our proposed AoA-based method. As only two APs were employed, as described in Section 4.1, we redesigned the RSSI range-based localization scheme as follows:

(1) The user held the smartphone and walked up or back toward the AP, ranging from 0 to 50 m, and recorded the measured RSSI at a step size of 0.5 m.
(2) The RSSI was fitted a function of the user’s distance to the AP, as illustrated in Figure 8.
(3) The user held the smartphone and stood facing the two APs in one of the positions $G_{1}$ to $G_{7}$, and recorded the RSSI values, corresponding to the $AP_{1}$ and $AP_{2}$.
(4) The distances ($R_{1}$ and $R_{2}$) to $AP_{1}$ and $AP_{2}$ were retrieved, based on the function obtained in (2).
(5) The user’s location could be estimated by solving the following set of equations:
  (23) $$\;\{\begin{array}{c} {\left(x-x_{1}\right)}^{2}+{\left(y-y_{1}\right)}^{2}=\;R_{1}{}^{2}\;\\ {\left(x-x_{2}\right)}^{2}+{\left(y-y_{2}\right)}^{2}=\;R_{2}{}^{2}\; \end{array}\;$$

It was obvious that the solutions to Equation (23) were the intersections of the two circles. Two circles were centered on $AP_{1}$ and $AP_{2}$, with $R_{1}$ and $R_{2}$ as the radius, respectively. If the intersection existed, there were usually two solutions to Equation (23), except for the special situation in which there was only one solution, when the two circles were tangent to each other (i.e., $\sqrt[{2}]{{\left(x_{2}-x_{1}\right)}^{2}+{\left(y_{2}-y_{1}\right)}^{2}}=R_{1}+R_{2}$).

**Figure 9:**
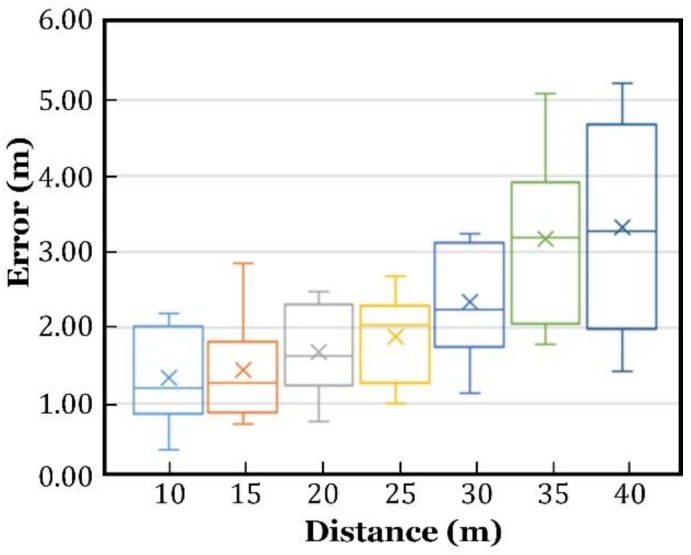
Indoor localization errors using the received signal strength indication (RSSI) range-based method.

In order to pick the right solution from the two, the blocking effect described in References [44,45] were utilized. As the human body can significantly attenuate Wi-Fi signals (both in 2.4 GHz and 5 GHz frequencies), the RSSI values on the smartphone would vary when the user held smartphone and spun at the location. As shown in Figure 8b, when the user lay between the AP and the smartphone, the direct path was severely blocked, resulting in a large attenuation of the RSSI. With the help of the blocking effect, additional direction information with regard to the APs was obtained, to determine the right solution. Localization was also performed for eight times, in each position, to obtain the statistical result, as plotted in Figure 9, and the comparison with our proposed AoA-based method is given in Table 2.

As shown in Figure 9, a similar phenomenon, as observed in Figure 7, occurred again when the localization accuracy decreased as the distance increased, despite the fluctuating minimum, maximum, median, and mean values of the location errors, which is more intuitional in Table 2. As both localization methods were essentially based on the RSSI information measured in a LOS-dominating environment, it was to be expected that they would have an equivalent location accuracy. However, compared to the solution of the binary quadratic equations in the RSSI range-based method, our proposed AoA-based method was much more efficient, as the calculation only involved a simple tangent function and basic arithmetic operations. Furthermore, the solution was unique in our method, even if only two APs were used for positioning.

### 4.4. Localization Accuracy

**Figure 10:**
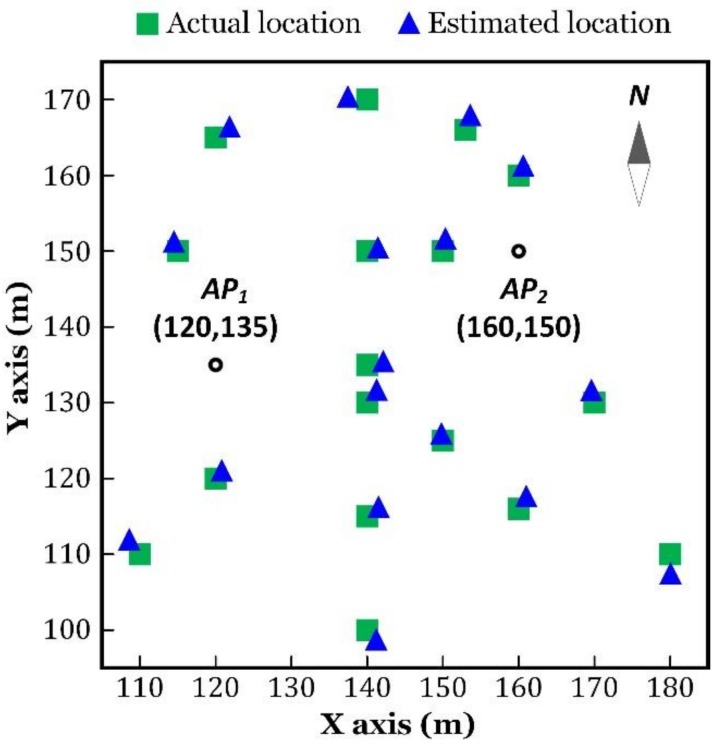
Indoor localization results.

**Figure 11:**
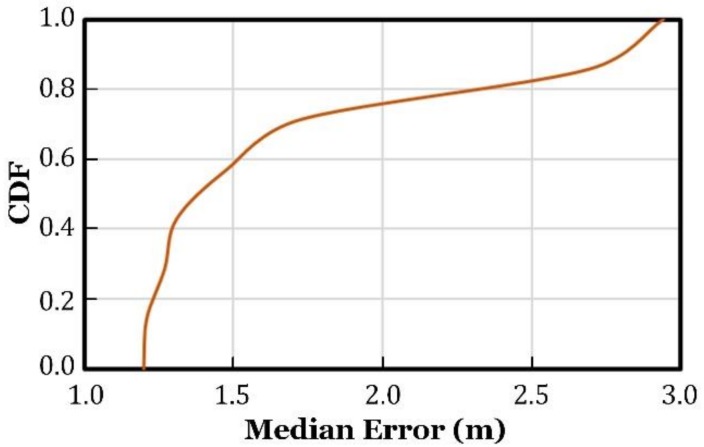
The cumulative distribution function (CDF) of localization error.

We tested the localization accuracy of our proposed algorithm in a more general environment, where the APs were randomly placed on the ceiling and the user wandered around the APs. The results are shown in Figure 10. Figure 11 plots the cumulative distribution function (CDF) of the median localization errors.

As shown in Figure 11, for over 80% of the test positions, localization error was less than 2.5 m.

## 5. Conclusions and Discussion

In order to provide room-level localization services for hospital outpatients using their own mobile devices, this paper proposed an efficient AoA-based wireless indoor localization algorithm that uses the coordinates and the angles of the arrival, provided by commercial Wi-Fi access points, to estimate the target’s position. Different from the available triangulation or trilateration localization algorithms, which utilize three or more APs and the complicated least square method, the proposed localization algorithm employs only two Aps, at a time, for positioning. All the possible positions in which the target might appear were comprehensively investigated, and the corresponding solutions were proven to be in the same form. Compared to the solution for the binary quadratic equations in the RSSI range-based method, the calculation in our proposed AoA-based method for positioning, only involves a simple tangent function and basic arithmetic operation, which could be efficiently performed on handheld mobile devices, such as smartphones. Two experiments were conducted to evaluate the feasibility and the localization performance. Experimental results showed that localization error was less than 2.5 m, about 80% of the time, in the LOS-dominating environments, which could satisfy the outpatients’ needs for wayfinding, in the hospital. 

However, the drawbacks were also obvious. The main disadvantage was that the infrastructure cost would be much higher to build a wireless local area network, using the Cisco 4800 series Aps, rather than deploying other conventional APs. The localization algorithm is also mainly designed for the LOS-dominating environments, which confines its application to a certain extent, in real practice. Therefore, further work could be done to improve this localization method. 

First, hybrid methods could be explored to tackle the positioning in the NLOS environments. For example, the AoA might be used in conjunction with a ToF. To be more specific, the phase difference between a pair of adjacent antennas is marked with a time stamp, by taking into account the relative flight time of their received incident radio waves. The AoA, which corresponds to the minimum ToF, could be considered to be the AoA representing the LOS path. 

Second, the time spent in positioning needs to be reduced for the sake of a better user experience. In other word, the user should be able to keep on walking at a normal pace during the positioning. The AoAs measurement and acquisition could be accelerated by developing specific applications to get rid of the manual operation.

Last, the APs’ deployment could be optimized to eliminate the blind points. For example, the APs could be staggered in the corridors or hall. When the target happens to be collinear with the two nearest APs, one of the AP should be replaced by another that is nearby.

## Figures and Tables

**Table 1:** Indoor localization results.

Position	Actual AoAs		Measured AoAs		Location		ErrorΔd
	$\;\alpha_{1}$	$\;\alpha_{2}$	$\;\alpha_{1}$	$\;\alpha_{2}$	Actual	Estimated	
$\;G_{1}$	17.7°	138.2°	20°	140°	(7.07, 7.07)	(5.92, 7.62)	1.27
$\;G_{2}$	22.5°	112.5°	25°	115°	(10.61, 10.61)	(10.65, 9.33)	1.28
$\;G_{3}$	25.9°	93.4°	25°	95°	(14.14, 14,14)	(13.82, 13.44)	0.77
$\;G_{4}$	28.4°	81.4°	30°	80°	(17.68, 17.68)	(18.40, 19.28)	1.76
$\;G_{5}$	30.3°	73.7°	33°	77°	(21.21, 21.21)	(20.29, 22.92)	1.94
$\;G_{6}$	31.9°	68.5°	34°	70°	(24.75, 24.75)	(24.76, 26.82)	2.07
$\;G_{7}$	33.2°	64.8°	36°	68°	(28.28, 28.28)	(27.47, 30.85)	2.70
$\;G_{8}$	106.3°	149.6°	105°	150°	(−21.21, 21.21)	(−20.49, 20.49)	1.02
$\;G_{9}$	253.7°	210.4°	256°	210°	(−21.21, −21.21)	(−20.04, −20.23)	1.52
$\;G_{10}$	329.5°	286.3°	330°	286°	(21.21, −21.21)	(20.95, −20.76)	0.52

**Table 2:** Indoor localization results.

Position	Location Error Using Our Proposed Method				Location Error Using RSSI Range-Based Method			
	Max	Min	Mean	Median	Max	Min	Mean	Median
$\;G_{1}$	1.87	0.10	1.27	1.27	2.18	0.38	1.33	1.20
$\;G_{2}$	1.35	0.26	1.05	1.30	2.85	0.72	1.43	1.26
$\;G_{3}$	1.76	0.17	1.10	1.21	2.47	0.75	1.67	1.62
$\;G_{4}$	2.24	0.27	1.49	1.73	2.68	1.00	1.87	2.04
$\;G_{5}$	2.68	0.52	1.54	1.48	3.24	1.13	2.33	2.23
$\;G_{6}$	3.33	0.50	2.30	2.69	5.09	1.77	3.16	3.18
$\;G_{7}$	3.29	0.42	2.40	2.94	5.22	1.42	3.31	3.26
